# Comparative Cytotoxicity of Menthol and Eucalyptol: An In Vitro Study on Human Gingival Fibroblasts

**DOI:** 10.3390/pharmaceutics16040521

**Published:** 2024-04-09

**Authors:** Clara Puig-Herreros, José Luis Sanz, David García-Bernal, Francisco Javier Rodríguez-Lozano, Laura Murcia, Leopoldo Forner, James Ghilotti, Ricardo E. Oñate-Sánchez, Sergio López-García

**Affiliations:** 1 Speech Therapy University Clinic, Department of Basic Psychology, Universitat de València, 46010 Valencia, Spain; 2 Departament d’Estomatologia, Facultat de Medicina i Odontologia, Universitat de València, 46010 Valencia, Spainsergio.lopez-garcia@uv.es (S.L.-G.); 3 Department of Biochemistry, Molecular Biology B and Immunology, Faculty of Medicine, University of Murcia, 30120 Murcia, Spain; david.garcia23@um.es; 4 Biomedical Research Institute (IMIB), 30120 Murcia, Spain; 5 Department of Dermatology, Stomatology, Radiology and Physical Medicine, Morales Meseguer Hospital, Faculty of Medicine, University of Murcia, 30008 Murcia, Spain; 6 Department of Health Sciences, Catholic University San Antonio of Murcia, 30107 Murcia, Spain

**Keywords:** eucalyptol, menthol, cytotoxicity, gingival fibroblasts

## Abstract

The aim of this study was to assess the influence of eucalyptol and menthol on the cell viability, migration, and reactive oxygen species production of human gingival fibroblasts (GFs) in vitro. Three different concentrations of eucalyptol and menthol were prepared following ISO 10993-5 guidelines (1, 5, and 10 mM). GFs were isolated from extracted teeth from healthy donors. The following parameters were assessed: cell viability via MTT, Annexin-V-FITC and 7-AAD staining, and IC_50_ assays; cell migration via horizontal scratch wound assay; and cell oxidative stress via reactive oxygen species assay. Data were analyzed using one-way ANOVA and Tukey’s post hoc test. Statistical significance was established at *p* < 0.05. Eucalyptol and Menthol exhibited high cytotoxicity on gingival fibroblasts, as evidenced by cytotoxicity assays. Eucalyptol showed lower levels of cytotoxicity than menthol, compared to the control group. The cytotoxicity of the tested substances increased in a concentration-dependent manner. The same occurred in a time-dependent manner, although even 10 min of exposure to the tested substances showed a high cytotoxicity to the GFs. Commercially available products for oral application with these substances in their composition should be tested for cytotoxicity before their use.

## 1. Introduction

Menthol, (1R,2S,5R)-2-isopropyl-5-methylcyclohexanol or C10H20O, is a monoterpene, an alcohol existing in peppermint that is found in a solid state. It is used in toothpastes, mouthwashes, chewing gums, candies, inhalers, analgesic pastes, creams, gels, and lotions. In these presentations, it can be used as a therapeutic agent or for other purposes. For example, it can be used as a flavoring agent in the coadjuvant and symptomatic treatment of oral pathology and the upper respiratory tract (pharynx and larynx). This is because mentha piperita essential oil, in which menthol is found at 30.69% [1], is associated with antibacterial, anti-inflammatory and antioxidant activity [1,2,3].

It has also been applied for the treatment of vaginal infections, cold sores, and alopecia. Menthol causes a sensation of coolness due to the stimulation of sensory neurons, especially through the transient receptor potentials TRPM8 and TRPA1, increasing the intracellular calcium content. This results in a perception of cold and an analgesic action through the excitation of GABA receptors and sodium ion channels [4].

Similarly, menthol has a possible cytotoxic action. Its activity against different tumor cell lineages has been investigated [1,4,5], including some related to cervical cancer [6] or against ovarian cancer cells [7] and rat glioma cells. Menthol also inhibits murine leukemia cells [8], but shows low toxicity in colorectal cancer cells [3]. It is clearly cytotoxic to melanoma cells by acting on the TRPM8-transient receptor potential melastatin 8-pathway [9].

In this regard, the antitumor action of drugs such as paclitaxel and vincristine is enhanced by menthol on human hepatocellular carcinoma cells without eliciting cytotoxic activity [10]. However, menthol makes this tumor resistant to anticancer drugs such as epirubicin or cisplatin by inducing MRP2, or multidrug resistance associated protein 2 [11]. Menthol potentiates the action of other active principles, such as puerarin (an isoflavone glycoside that possesses anti-inflammatory and antimicrobial properties), by facilitating its transport through increased cell membrane permeability [12]. The genotoxicity of menthol has also been investigated. It induces, in a weak manner, aberrations in Chinese hamster ovary cells [13].

Eucalyptol, or 1,8 cineol (C10H18O), is an epoxy-monoterpene extracted from eucalyptus and other plants. It is also used in upper airway pathology [14] due to its anti-inflammatory action [15]. It is found in liquid form and is used in inhalers, mouthwashes, and pastes for dermatitis, nasal congestion, and throat problems. Eucalyptol shows an antifungal effect, but this is associated with toxic effects [16]. However, it has been proposed for use in nanofibers for the treatment of Candida infections [17]. Recently, its antiviral action against COVID-19, applied as a nebulized nano-emulsion, was described [18].

The cytotoxic action of eucalyptol on tumor cells has been described in mouse lymphoma cells [19], in human colon cancer cells [14], in breast cancer cells [20], in colorectal cancer cells [21], and in hamster ovarian cells [22]. In the oral setting, used in endodontic dental treatments, it shows cytotoxicity against periodontal ligament stem cells in a concentration-dependent manner [23]. Rosemary oil (Rosmarinus officinalis), which contains eucalyptol, an α-pinene (bronchodilator), and L-camphor or C10H16O (terpenoid with mild analgesic action), shows significant cytotoxicity on colorectal cancer cells with low antimicrobial action [3]. Cytotoxicity on non-cancer cells (HaCaT) was not significant for either menthol or eucalyptol [3].

Eucalyptol is also slightly genotoxic because it exerts a concentration-dependent oxidative effect on DNA [14], although at low concentrations it facilitates DNA repair mechanisms [21]. Other research reports no genotoxicity [19,22].

Regarding their application in the oral cavity, an issue concerns their use as flavorings. For example, in electronic cigarettes, menthol is used as a flavoring agent at >1 mg/mL. It has been described that the risk of cancer would be decreased by using pure menthol and not peppermint oil [24]. Generally, e-cigarettes induce inflammation in epithelial cells and lung fibroblasts, which inhibits wound healing capacity [25]. Menthol present in cigarette refills can be 30 times higher than the cytotoxic concentration [26]. Menthol also increases cytotoxicity in A549 alveolar epithelial cells [27]. Small cigarettes, both flavored and unflavored, produce similar levels of cytotoxicity on lung epithelial cells [28]. There is menthol-induced toxicity in mouse lung cells [29]. Menthol and other flavorings may also be partially responsible for the cytotoxicity observed on bronchial epithelial cells from electronic cigarettes that also contained nicotine [30].

Their effects on other tissues have also been analyzed. Menthol-flavored e-cigarette liquids significantly induce cell death (IC_50_: 1.45 ± 0.14%) in middle ear epithelial cells and may be a risk factor for the development of otitis media [31]. There is also a synergism between nicotine and menthol that increases their cytotoxicity in retinal pigment epithelial cells, so they would also constitute a possible risk factor for retinal pathology [32]. Lastly, the cytotoxicity of e-cigarettes does not appear to be directly dependent on menthol—neither liquid nicotine nor ROS formation—on human vascular endothelial cells [33].

To our knowledge, the effect of these products on tissues in the oral cavity has not been studied in depth. Accordingly, the aim of our study is to extend the knowledge on the effect of menthol and eucalyptol on oral cells, specifically gingival fibroblasts, by determining their biocompatibility or cytotoxicity. We hypothesize that both menthol and eucalyptol are not cytotoxic to gingival fibroblasts.

## 2. Materials and Methods

This manuscript has been written in accordance with the following reporting guidelines: “Guidelines for reporting pre-clinical in vitro studies on dental materials” [34].

### 2.1. Cell Isolation and Culture

The cell extraction methodology was approved by the Human Research Ethics Committee of the Universidad de Murcia, with the identifier 3686/2021. This involved collecting gingival tissue from ten healthy subjects scheduled for dental extractions who had consented in compliance with the Helsinki Declaration. The procedure commenced with the mechanical mincing of the gingival tissues using a scalpel, followed by cleansing in Phosphate-Buffered Saline (PBS) and a 1% penicillin/streptomycin (P/S) solution (Invitrogen, Paisley, Scotland). Subsequently, the tissues underwent enzymatic digestion in a serum-free Dulbecco’s Modified Eagle Medium (DMEM) (Gibco, ThermoFisher Scientific, Waltham, MA, USA), enriched with 0.2% dispase II (Gibco) and 0.1% collagenase A (Roche Diagnostics, Basel, Switzerland), at 37 °C for 2 h. Post-digestion, the human gingival fibroblasts (GFs) were rinsed, filtered through 100-µm nylon strainers (BD Biosciences, San Jose, CA, USA), and cultured in DMEM supplemented with 10% fetal bovine serum (Sigma-Aldrich, St. Louis, MO, USA), 1% GlutaMAXTM (Gibco), and 1% P/S, under 5% CO_2_ at 37 °C. Once the GFs achieved 70–80% confluence, they were detached using 0.25% TrypLE Express (Gibco) and sub-cultured for use in experiments from the 2nd to the 5th passages. On 6-well plates, 3 × 10^3^ cells were seeded in growth medium and left to adhere for 24 h to be used for the assays.

### 2.2. Sample Preparation

The studied substances were Eucalyptol (99% minimum concentration; DentaFlux, Madrid, Spain; batch no: 010921) and Menthol (99% minimum concentration; Sigma-Aldrich, St. Louis, MO, USA; batch no: 1003590440).

For the cellular assays, sample dilutions were obtained from the tested substances under sterile conditions. Substance eluates were formulated following ISO 10993-5 guidelines. Substance dosage was calculated in millimolar (mM) due to the solid state of Menthol and the liquid state of Eucalyptol. First, basal dilutions of 2.5 M of eucalyptol and menthol in ethanol were prepared. Then, eluates were further diluted in DMEM (Gibco) and filtered through a 0.22-µm syringe filter to produce the following dilutions: 25, 12.5, 6.25, 3.12, 1.5, 0.7, 0.39 y, 0.195 mM. Lastly, based on the results of the IC_50_ assay (described below), three different dilutions were prepared for the rest of the cellular assays: 1, 5, and 10 mM.

### 2.3. IC_50_ Assay

Cell samples were treated with different concentrations (25, 12.5, 6.25, 3.12, 1.5, 0.7, 0.39, or 0.195 mM) of the tested substances (menthol or eucalyptol). Two conditions were assessed: 10 min of exposure to the different eluates of Menthol or Eucalyptol, or exposure for the duration of the assay. In the first condition (10 min of exposure), samples were washed after the exposure. In both conditions, samples were cultured for 24 h. Cell metabolic activity was measured by means of an initial MTT assay, using the same methodology as the final MTT assay described in Section 2.5. To assess their cytotoxicity, the concentrations of the studied solvents that could decrease cell viability by 50% after 0 and 24 h (half maximal inhibitory concentration (IC_50_)) were assessed graphically by plotting the percentage of metabolic activity on the Y-axis and the concentration in mM of each substance on the X-axis. Furthermore, IC_50_ values were analyzed by non-linear regression using GraphPad Prism software version 8.1.0 (GraphPad Software Inc., San Diego, CA, USA).

### 2.4. Cell Apoptosis/Necrosis Assay: Annexin-V-FITC and 7-AAD Staining

GF viability was quantified after 72 h of culture at 37 °C in complete growth medium (control) or in complete growth medium exposed to 1, 5, or 10 mM of the tested substance eluates for 10 min or 72 h. To evaluate cell viability, the application of Annexin-V-FITC and 7-AAD staining techniques (sourced from BD Biosciences, San Jose, CA, USA) was performed in strict adherence to the supplied protocols by the manufacturer. Analysis of the stained specimens was conducted within an hour using flow cytometry, specifically employing the FACS Calibur Flow cytometer provided by Becton Dickinson (Franklin Lakes, NJ, USA). The quantification of cell viability was expressed as a percentage, with categorization into distinct stages: viable cells were identified by the absence of both stains (double negative), early apoptotic cells were marked by the presence of Annexin-V-FITC alone (7-AAD negative), and cells in late apoptosis or necrosis were indicated either by 7-AAD positivity in the absence of Annexin-V-FITC or by positivity for both stains, respectively.

### 2.5. MTT Assay

An evaluation of the cytotoxic effects exerted by three concentrations (1, 5, or 10 mM) of eluates obtained from Menthol or Eucalyptol on GFs was conducted. This assessment was carried out in comparison with GFs incubated in an unconditioned growth medium, which served as the negative control group. The methodology employed for this evaluation was the 3-(4,5-dimethylthiazol-2-yl)-2,5-diphenyltetrazolium bromide (MTT) assay, a standardized technique for measuring cell metabolic activity as an indirect marker of cell viability and cytotoxicity. Briefly, GFs were seeded onto 96-well plates with 180 μL of DMEM and incubated for 24 h at 37 °C, 5% CO_2_, and 95% humidity. The substance eluates were introduced into the culture medium with 1 × 10^4^ GFs (*n* = 3 per test group). MTT reagent (Sigma Aldrich, USA) was applied for 4 h according to the manufacturer’s instructions. To facilitate the solubilization of formazan crystals generated by the metabolic activity of viable cells following the reduction of the MTT reagent, the plates were maintained under dark conditions for a duration of 4 h. Subsequent to the observation of a purple precipitate, dimethyl sulfoxide (DMSO; Sigma-Aldrich, USA) was administered to each well in a volume of 100 μL. This was followed by a brief agitation of the plates, ranging from 1 to 5 min, to ensure complete dissolution of the precipitate. The absorbance at a wavelength of 570 nm for each well was subsequently measured using a microplate reader (ELx800; Bio-Tek Instruments, Winooski, VT, USA) at three different time points: 24, 48, and 72 h post-culture. Two conditions were assessed: 10 min of exposure to the different eluates of Menthol or Eucalyptol, or exposure for the duration of the assay (24, 48, and 72 h).

### 2.6. Horizontal Scratch Wound Assay

A horizontal wound healing assay was carried out to assess the migration ability of GFs in response to the three different concentrations of menthol and eucalyptol (1, 5, or 10 mM). A comparison was made with cells cultured in unconditioned growth medium (negative control group). In summary, GFs were allocated into 6-well plates at a density of 2 × 10^5^ cells per well, with three replicates for each substance under investigation as well as the control group. The cells were cultured until a confluent monolayer was established. Subsequently, a standardized superficial horizontal scratch was introduced across each cell monolayer utilizing a sterilized 200 μL pipette tip. Following this, each well underwent triple rinsing to eliminate any detached cell fragments. The migration of GFs across the scratch wound was monitored using an optical microscope (Olympus, Tokyo, Japan). Photographic documentation was conducted at 24, 48, and 72 h post-injury for each test substance and the control group to evaluate the dynamics of cellular migration and wound closure. Again, two conditions were assessed: 10 min of exposure to the different eluates of Menthol or Eucalyptol, or exposure for the duration of the assay (24, 48, and 72 h). ImageJ software v1.48 (National Institutes of Health, Bethesda, MD, USA) was used to measure the percentage of open wound area at each time point relative to the same wound area at 0 h in the same well.

### 2.7. Intracellular ROS Measurement

The quantification of intracellular reactive oxygen species (ROS) levels within GFs subjected to treatment with three distinct concentrations of menthol and eucalyptol (1, 5, or 10 mM) was conducted via flow cytometry. This analysis employed the general oxidative stress marker 5-(and-6)-chloromethyl-2′,7′-dichlorodihydrofluorescein diacetate (CM-H2DCFDA) (Molecular Probes, Eugene, OR, USA). Initially, GFs were dissociated using TrypLE Express dissociation reagent (Thermo Fisher Scientific, Waltham, MA, USA), followed by a double rinse with DPBS (Gibco, Waltham, MA, USA). Subsequently, the cells were incubated with 5 μm CM-H2DCFDA in darkness for 30 min at 37 °C. Intracellular fluorescence was then quantified using a LSR Fortessa X-20 flow cytometer (Becton Dickinson, Franklin Lakes, NJ, USA), employing an excitation wavelength of 492 nm and an emission wavelength of 517 nm. Analysis of the CM-H2DCFDA-positive cells was performed utilizing FlowJo v10 software (FlowJo LLC, Ashland, OR, USA), facilitating a comprehensive assessment of oxidative stress levels induced by the treatments.

### 2.8. Statistical Analysis

For each substance (Menthol and Eucalyptol), experimental conditions and measurements were replicated three times to ensure reliability. The results were articulated as means accompanied by standard deviations (SD) to provide a clear representation of data variability. Prior to conducting further analyses, a Q-Q plot was utilized to ascertain the normality of the data distribution, ensuring the appropriateness of subsequent statistical tests. Statistical evaluations were conducted employing either one-way or two-way Analysis of Variance (ANOVA), complemented by Tukey’s post hoc test for detailed pairwise comparisons, utilizing GraphPad Prism software version 8.1.0 (GraphPad Software, San Diego, CA, USA). For the application of one-way ANOVA, the data were categorized based on the time intervals of observation (24, 48, and 72 h) and analyzed discretely to discern temporal patterns in the responses. Each dilution/eluate was considered an independent experimental condition. Statistical significance was set at *p* < 0.05.

## 3. Results

### 3.1. IC_50_ Assay

**Figure 1:**
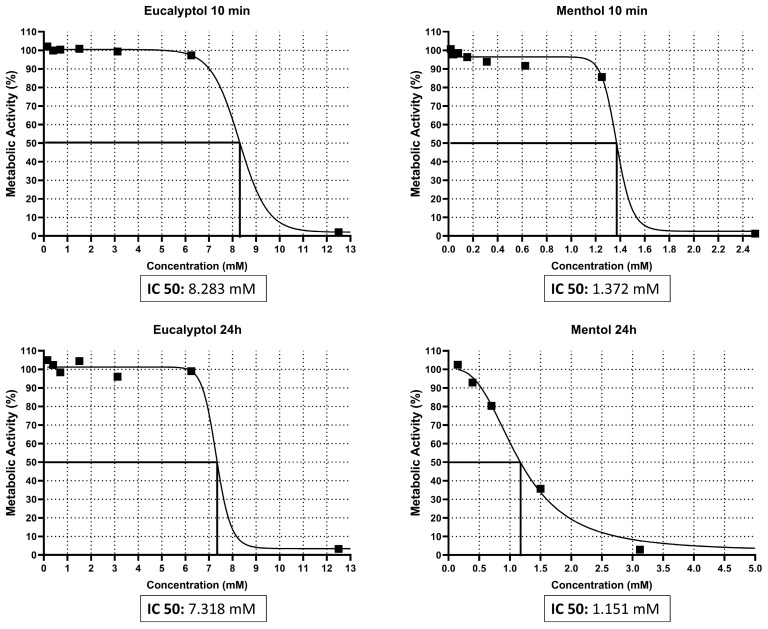
Results from the IC_50_ assay. The data are illustrated graphically by plotting the percentage of metabolic activity on the y-axis and the concentration percentage of each solvent on the x-axis. Squares represent each of the data points.

The IC_50_ values (i.e., the percentage concentration of each solvent to inhibit 50% of GF viability) were, after 10 min or 24 h of exposure: Eucalyptol = 8.283 mM and 7.318 mM, respectively; Menthol = 1.372 mM and 1.151 mM, respectively (Figure 1). Thus, the following concentrations were selected for the following cellular assays: 1, 5, and 10 mM.

### 3.2. Cell Apoptosis/Necrosis Assay: Annexin-V-FITC and 7-AAD Staining

**Figure 2:**
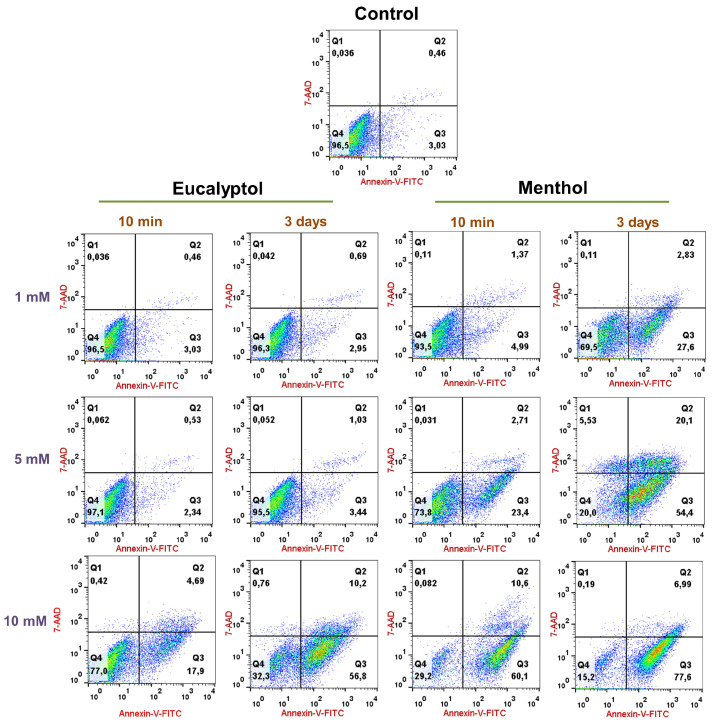
Results from the cell apoptosis/necrosis assay. Numbers inside representative dot plots represent percentages of live (Q4 quadrants), early apoptotic (Q3 quadrants), and late apoptotic and necrotic cells (Q1 and Q2 quadrants).

Cell viability rates of GFs exposed to different concentrations (1, 5, or 10 mM) of the tested substances (Eucalyptol or Menthol) after 10 min or 72 h of culture are presented in Figure 2. The exposure of each of the concentrations of tested substances for 3 days resulted in lower cell viability rates in all cases compared to the exposure for 10 min. Additionally, the increase in dosage from 1 to 5 mM and from 5 to 10 mM resulted in lower cell viability rates in all cases. All samples maintained their predominant cell viability rate in Q4 (viable (double negative), except for GFs submitted to 3 days of exposure to 10 mM Eucalyptol, 3 days of exposure to 5 mM Menthol, or 10 min or 3 days of exposure to 10 mM Eucalyptol. In all cases, cell viability rates were higher in GFs exposed to Eucalyptol than to Menthol, which remained predominantly at Q3 (Annexin-V-FITC positive, 7AAD negative). At the same time, all cell viability rates of GFs exposed to the tested substances were lower than the control group. Altogether, results from the cell apoptosis/necrosis assay reveal concentration-dependent and exposure-time-dependent cytotoxicity from Eucalyptol and Menthol, which was higher in all cases in eucalyptol.

### 3.3. MTT Assay

**Figure 3:**
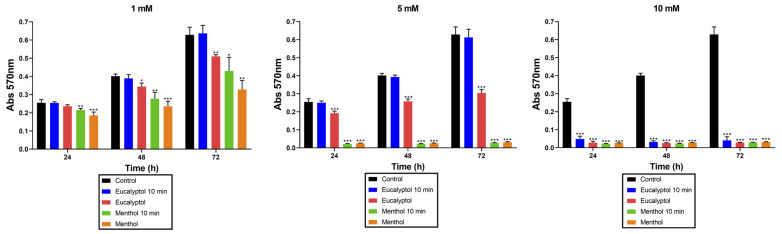
Results from the MTT assay. Data are presented as absorbance values (570 nm) at 24, 48, and 72 h of culture of GFs exposed to the tested substance eluates (1, 5, or 10 mM) for 10 min or for the duration of the assay, compared to the control (* *p* < 0.05; ** *p* < 0.01; *** *p* < 0.001).

MTT assay results of GFs exposed to different concentrations (1, 5, or 10 mM) of the tested substances (Eucalyptol or Menthol) for 10 min or for the duration of the assay and cultured for 24, 48, and 72 h are presented in Figure 3. All the tested concentrations exhibited significantly higher cytotoxicity than the control group (GFs cultured in growth medium without substance eluates) at all measured time points, except for 10 min exposure to 1 mM or 5 mM of Eucalyptol, where the difference with the control group was not significant. Again, the lower the concentration of the tested substances, the higher the cell viability in all cases. In addition, the exposure of each of the concentrations of tested substances for 3 days resulted in lower cell viability in all cases compared to the exposure for 10 min.

### 3.4. Horizontal Scratch Wound Assay

**Figure 4:**
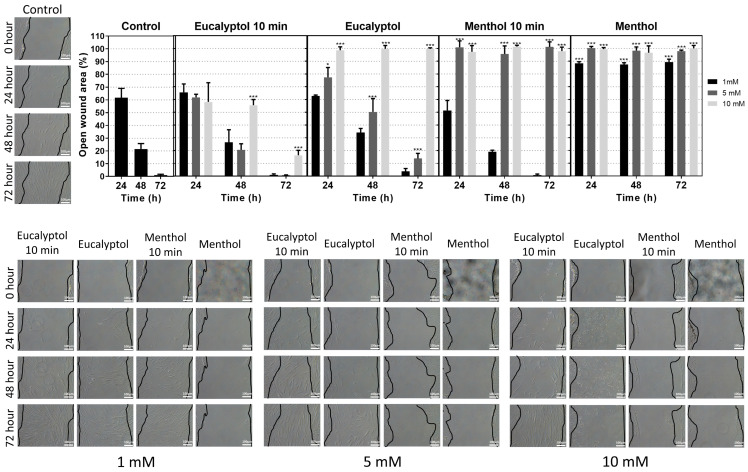
Results from the horizontal scratch wound assay. Graphical results are presented as mean relative wound closure (RWC) percentages at each culture duration (24, 48, or 72 h) and exposure to the tested substance eluates (1, 5, or 10 mM) for 10 min or for the duration of the assay, relative to the total wound area at 0 h. Asterisks designate significant differences compared to the control (* *p* < 0.05, *** *p* < 0.001).

The results of the migration ability of GFs exposed to substance eluates are presented in Figure 4. A total of 24, 48, and 72 h of exposure to all the concentrations of Menthol resulted in a significantly greater open wound area compared to the control group. The same occurred with 10 min of exposure and 24, 48, and 72 h of culture with 5 and 10 mM of Menthol. Only the culture of GFs with 5 and 10 mM of Eucalyptol for 24, 48, and 72 h resulted in significantly greater open wound areas than the control group. Similarly, only the 10 min exposure of GFs to 10 mM of Eucalyptol and culture for 48 and 72 h of culture resulted in significantly greater open wound areas. Again, the exposure of each of the concentrations of tested substances for 3 days resulted in a greater percentage of open wound area in all cases compared to the exposure for 10 min.

### 3.5. Intracellular ROS Measurement

**Figure 5:**
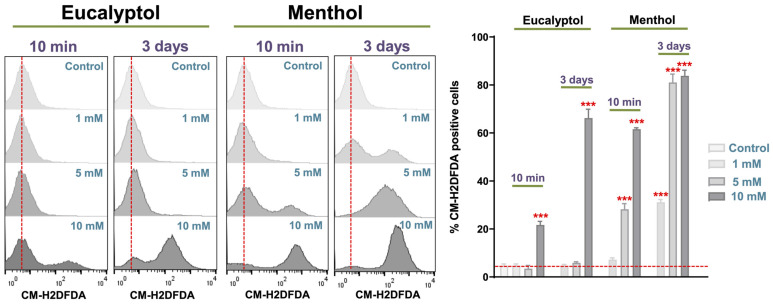
Results from the intracellular ROS measurement assay on the percentage of CM-H2DCFDA-positive cells after exposure to the tested substance eluates (1, 5, or 10 mM) for 10 min or 72 h, compared to the control group (*** *p* < 0.001).

The intracellular ROS production of GFs treated with several concentrations of the different substances (1, 5, or 10 mM) for 10 min and 72 h is shown in Figure 5. Three days of exposure to all the concentrations of Menthol resulted in significantly greater intracellular ROS compared to the control group. The same occurred with 10 min of exposure to 10 and 5 mM of Menthol. Only the culture of GFs with 10 mM of Eucalyptol for 10 min or 3 days resulted in significantly greater intracellular ROS than the control group. Again, the exposure of each of the concentrations of tested substances for 3 days resulted in greater intracellular ROS in all cases compared to the exposure for 10 min.

## 4. Discussion

In many cases, the oral cavity is the route of entry for different forms of presentation of products that, with or without therapeutic purposes, contain menthol or eucalyptol. Previous evidence has highlighted the cytotoxicity of these substances in various cell lineages and concentrations [1,5,14,19]. However, evidence regarding their biological effects in the oral cavity remains limited. Thus, the aim of this in vitro study was to assess the cytotoxicity of menthol and eucalyptol on gingival fibroblasts.

For the cytotoxicity assays, gingival fibroblasts were used as the target cell population. Their use for the in vitro analysis of the biological properties of materials that contact the oral cavity is extended in the literature [35,36]. With regard to sample characteristics, various concentrations of the tested substances were used (1, 5, and 10 mM), as performed in previous in vitro studies with similar methodology [23,37]. The justification for the use of different concentrations, based on the results of the IC_50_ assay, lies in the fact that different proportions of menthol or eucalyptol may contact oral tissues depending on their application format.

Regarding the cytotoxicity assessment, a series of assays were performed: a MTT assay to determine the cellular metabolic activity as a means of cell viability analysis; an Annexin-V-FITC and 7-AAD staining assay to complement the cell viability assay; a horizontal wound healing assay to analyze cell migration ability; and a measurement of intracellular reactive oxygen species to assess cell oxidative stress. The selection of these methods to assess the cytotoxicity of the tested substances was based on previous studies with a similar objective [11,23,38,39]. Other cytotoxicity assays are available. For example, a trypan blue assay can also be performed to assess cell viability, as performed in previous studies [22,37]. However, it has been reported that the MTT assay is the most sensitive and offers major advantages in terms of speed, simplicity, and precise quantitation [40].

Standardization of the methodology of in vitro studies from the same field of research is crucial for their collective assessment and contrast. For this reason, in the present study, sample preparation was performed following ISO 10993-5 guidelines (tests for in vitro cytotoxicity), as performed in previous similar studies [23,35]. Similarly, standardized reporting guidelines were followed throughout this manuscript. A modified CONSORT checklist for in vitro studies on dental materials was followed [34] to overcome the absence of specific guidelines. Reporting guidelines for in vitro studies on human cell lineages should be developed in the future.

Among the limited available evidence regarding the biological effects of eucalyptol or menthol in the oral cavity, a recent study reports the production of inflammatory cytokines in gingival epithelial cells with the use of nicotine pouches in which menthol is used as a flavoring [41]. In the present study, both menthol and eucalyptol exhibited significant cytotoxicity on gingival fibroblasts. However, this cytotoxicity was found to be concentration-dependent, in a way that the lower the concentration of the tested substances, the lower the cytotoxicity. Specifically, the highest dilutions of eucalyptol and menthol (1 mM) exhibited comparable results to those of the control group (gingival fibroblasts cultured in unconditioned medium) in terms of cytotoxicity in most cases. This tendency was also reported in a previous study on the cytotoxicity of eucalyptol as a solvent for root canal treatment on human periodontal ligament stem cells [23] and peritoneal macrophages [37]. Concentration-dependent cytotoxicity was observed recently for menthol on leukemic cell lines [38].

Eucalyptol, on the other hand, is recognized as an anti-inflammatory compound but exhibited significantly higher ROS production in its higher concentration (10 mM). The anti-inflammatory effect of eucalyptol may be concentration-dependent, in a way that small dosages may present anti-inflammatory properties while an increase in its concentration above a certain threshold may be detrimental for cells. This has been observed in other compounds with biomedical applications [42,43]. A possible explanation for the high ROS production from the tested substance may be its cytotoxicity at higher concentrations. ROSs are byproducts of cellular metabolism. Their release or production in higher quantities is indicative of cell oxidative stress, which indicates cell damage [44].

In a similar manner, the longer the exposure time of gingival fibroblasts to menthol or eucalyptol, the higher their cytotoxicity. Specifically, 10 min of exposure to the tested substances led to higher cell viability and migration and a lower release of reactive oxygen species than exposure to the whole duration of the respective cytotoxicity assays. The time-dependent cytotoxicity of menthol has also been described in previous studies on different cell lineages, such as human osteoblasts and murine fibroblasts [45].

Nonetheless, conclusions on the specific concentrations, the threshold from which eucalyptol or menthol become cytotoxic to oral cells or the time of exposure required for them to exhibit detrimental effects on oral cells cannot be drawn from the results of this study. This acts as the main limitation of this study, which was performed in controlled laboratory conditions on a specific cell line. Instead, the results should be interpreted as preliminary evidence, which highlights the importance of the cautious use of menthol and eucalyptol in products that contact the oral tissues and calls for further evidence in this regard. At the same time, considering the previous recent evidence on the potential therapeutic use of the cytotoxicity of eucalyptol and menthol in the treatment of tumoral cells [20,39,46], this study acts as supporting evidence in that regard.

Lastly, it should be highlighted that eucalyptol showed lower levels of cytotoxicity compared to the control group than menthol, as evidenced by the cell viability, cell migration, and reactive oxygen species release assays. To our knowledge, there is no previous evidence on the comparative cytotoxicity of eucalyptol and menthol. Nonetheless, a recent study assessed the cytotoxicity of a mouthwash containing both substances together with methyl salicylate and thymol on osteoblast-like cells and found alterations in their cell morphology and reduced cell viability, irrespective of the exposure time [47]. Again, this stresses the importance of assessing the safety and potential effects of common components in daily products that contact oral tissues before their use. However, further evidence is required to draw conclusions from the observed results.

## 5. Conclusions

Eucalyptol and Menthol exhibited high cytotoxicity on gingival fibroblasts, as evidenced by cell viability, apoptosis, migration, and reactive oxygen species production assays. Eucalyptol showed lower levels of cytotoxicity compared to the control group than menthol. The cytotoxicity of the tested substances increased in a concentration-dependent manner. The same occurred in a time-dependent manner, although even 10 min of exposure to the tested substances showed a high cytotoxicity to the GFs. Commercially available products for oral application with these substances in their composition should be tested for cytotoxicity before their use.

## Data Availability

The data presented in this study are available on request from the corresponding author.
